# Gait Kinematics Assessed by Vicon^®^ and Quality of Life Correlations in Multiple Sclerosis Patients: A Cross-Sectional Study

**DOI:** 10.3390/s25226909

**Published:** 2025-11-12

**Authors:** Ophélie Micolas, Marta Gil-Gregorio, Ane-Miren Uría-Oruezábal, Raúl López-González, Ángel González-de-la-Flor, María-José Giménez, María García-Arrabé, Cecilia Estrada-Barranco

**Affiliations:** 1 Department of Physiotherapy, Faculty of Medicine, Health and Sports, Universidad Europea de Madrid, 28670 Villaviciosa de Odón, Spain; ophelie.micolas@hotmail.fr (O.M.); marta.gil037@gmail.com (M.G.-G.); aneuria20@gmail.com (A.-M.U.-O.); angel.gonzalez@universidadeuropea.es (Á.G.-d.-l.-F.); maria.gararrabe@universidadeuropea.es (M.G.-A.); cecilia.estrada@universidadeuropea.es (C.E.-B.); 2 Department of Sports Sciences, Faculty of Medicine, Health and Sports, Universidad Europea de Madrid, 28670 Villaviciosa de Odón, Spain; raul.lopez@universidadeuropea.es

**Keywords:** multiple sclerosis, quality of life, gait kinematics, fatigue

## Abstract

Multiple sclerosis is an inflammatory and neurodegenerative disease that leads to motor, cognitive, and sensory impairments, significantly affecting walking and quality of life. This study aimed to analyze the relationship between quality of life and kinematic walking parameters in individuals with multiple sclerosis, as well as to evaluate the influence of fatigue, balance, and cognitive performance on different aspects of quality of life. A cross-sectional observational study was conducted with 32 patients diagnosed with multiple sclerosis with Expanded Disability Status Scale scores of ≤5.5. Quality of life was assessed using the MusiQoL questionnaire, and clinical variables included fatigue (Fatigue Scale for Motor and Cognitive Functions, Borg scale), balance (Berg Balance Scale), and cognitive performance (Trail Making Test). Walking kinematics were analyzed using the Vicon motion capture system to obtain walking speed, step frequency, and joint asymmetry indices. Spearman correlations and linear regression models were applied. Results showed significant correlations between quality of life and walking speed (rho = 0.506), step frequency (rho = 0.508), and knee asymmetry (rho = −0.525), as well as strong associations with cognitive fatigue (rho = −0.796) and balance (rho = 0.635). Regression models explained up to 58.4% of the variance in the Activities of Daily Living dimension. These findings indicate that quality of life in multiple sclerosis is influenced by both clinical and biomechanical factors, highlighting the importance of comprehensive assessments to guide physiotherapeutic interventions.

## 1. Introduction

Multiple sclerosis (MS) is a chronic, inflammatory, and neurodegenerative disease of the central nervous system (CNS) characterized by demyelination and axonal injury mediated by abnormal immune activation of T and B lymphocytes across a disrupted blood–brain barrier [1,2,3,4,5,6]. This condition primarily affects young adults between 20 and 40 years of age, with a higher incidence in women (approximately 3:1), and a rising global prevalence currently exceeding 2.5 million individuals [1,2,3,6,7,8,9]. Its etiology is multifactorial, involving genetic factors such as the HLADRB1*15:01 allele [10] and environmental contributors, including vitamin D deficiency, obesity, smoking, and Epstein–Barr virus infection [11,12,13,14]. This growing prevalence highlights the importance of developing objective and integrative assessment tools capable of linking neurological impairment with functional outcomes such as gait performance and quality of life.

The clinical presentation of MS is heterogeneous and is mainly classified into four phenotypes: relapsing–remitting MS (RRMS), primary progressive MS (PPMS), secondary progressive MS (SPMS), and progressive–relapsing MS (PRMS) [6,7,8]. RRMS accounts for more than 80% of initial cases and is characterized by reversible neurological relapses that progressively leave permanent sequelae. Approximately 50% of patients with RRMS convert to SPMS after 10–15 years [15]. Clinical manifestations include motor weakness, sensory disturbances, visual impairment, coordination deficits, fatigue, and cognitive dysfunction [16,17]. Progressive disability is commonly assessed using the Expanded Disability Status Scale (EDSS) [18,19].

One of the most critical aspects for functional independence is gait, a complex motor process requiring the coordinated integration of pyramidal, extrapyramidal, cerebellar, and vestibular systems along with proprioceptive inputs [20]. In individuals with MS, gait is impaired by muscle weakness, spasticity, cerebellar dysfunction, and sensory loss, resulting in asymmetrical patterns, reduced speed, and increased irregular cadence [20,21,22]. Previous studies indicate that up to 50% of patients experience walking difficulties in early stages [23], and nearly 50% require assistance for ambulation within the first 15 years [24]. Moreover, instability and balance disturbances contribute to an increased risk of falls, negatively impacting quality of life (QoL) [24,25,26,27].

Multiple investigations have demonstrated that factors such as fatigue—present in 75–95% of patients [28]—and reduced processing speed and executive function [29] directly affect functional independence, motor performance, and social participation. These factors compromise not only gait but also the subjective perception of physical, psychological, and social well-being, as reflected in questionnaires such as the Multiple Sclerosis International Quality of Life (MusiQoL31) [30,31]. In addition, biomechanical alterations in gait can be objectively quantified using three-dimensional motion capture systems such as Vicon^®^, which allow the measurement of kinematic parameters (e.g., speed, cadence, asymmetry indices) [32], and their correlation with clinical variables to identify functional predictors and guide physiotherapeutic interventions [33,34,35,36]. In this context, Tsiakiri et al. [37] emphasize the integration of advanced technological approaches to optimize personalized rehabilitation strategies, enhance early diagnostic accuracy, and enable continuous long-term monitoring of disease progression in clinical practice.

Previous studies have reported that reduced gait velocity and cadence are associated with greater functional limitation and poorer QoL in individuals with MS [38]. These findings suggest that gait performance is a relevant indicator of daily functioning and well-being. However, most research has analyzed these parameters in isolation, without integrating three-dimensional kinematic variables with multidimensional measures of QoL and related clinical factors such as fatigue, balance, or cognition [39].

Based on this evidence, the present study aimed to analyze the relationship between QoL and kinematic gait parameters in individuals with MS, and to explore the influence of fatigue, balance, and cognitive performance on different dimensions of QoL, in order to identify clinically and biomechanically relevant predictors for physiotherapy practice.

## 2. Materials and Methods

An observational cross-sectional study involving patients diagnosed with MS was performed in the Human Performance Research Laboratory of Universidad Europea de Madrid (Villaviciosa de Odón, Madrid, Spain). The study protocol (ECL.24/753-E_Tesis) was approved by the Research Ethics Committee of Hospital Clínico San Carlos (Madrid, Spain). The study was conducted in accordance with the principles of the Declaration of Helsinki [40], and Spanish legislation regarding personal data protection and guarantee of digital rights [41]. All participants received written and verbal information about the study and provided informed consent prior to participation. Participants who requested it received an individual gait report; overall study results will be shared after publication.

An a priori power estimation for the main correlation analyses (two-tailed, Fisher’s z approximation; equivalent to G*Power’s (version 3.1.9.2) Correlation: Bivariate normal model) assuming an anticipated effect size of ρ = 0.50, α = 0.05, and 1–β = 0.80 indicated a minimum of 30 participants. The final sample (n = 32), therefore, provided adequate power (achieved power ≈ 0.84 for ρ = 0.50).

Recruitment was conducted via social media advertisements and by distributing study information to MS associations and foundations in the Autonomous Community of Madrid. All participants had a confirmed diagnosis of MS according to the revised McDonald criteria [42,43,44], were between 18 and 65 years of age, and had an Expanded Disability Status Scale (EDSS) score of ≤5.5, which ensured functional ambulation, with or without the use of assistive devices, and without the need for rest over approximately 100 m [45]. Additional inclusion criteria included the ability to comprehend and follow study instructions and to provide written informed consent. Exclusion criteria included the inability to access the research center, a relapse of MS within the month prior to enrollment, and a history of previous trauma, disorders, or comorbidities that could affect movement, gait, or balance. Patients with severe or unstable pulmonary or cardiac disease, conditions inherently associated with fatigue such as hypothyroidism, severe depression, or cardiorespiratory disorders, or severe visual impairment were also excluded [46,47].

### 2.1. Study Variables

For the analysis, the MusiQoL-31 questionnaire was considered overall (all 31 items) and subdivided into its nine distinct dimensions [30]: Activities of daily living (ADL; items 1 to 8), Psychological well-being (PWP; items 9 to 12), Symptoms (SPT; items 13 to 16), Relationships with friends (RFr; items 17 to 19), Relationships with family (RFa; items 20 to 22), Sentimental and sexual life (SSL; items 23 and 24), Coping (COP; items 25 and 26), Rejection (REJ; items 27 and 28), and Relationship with the healthcare system (RHCS; items 29 to 31).

Gait analysis was performed using a Vicon^®^ Motion Capture System (Vicon Motion Systems Ltd., Oxford, UK), one of the most widely validated technologies for three-dimensional biomechanical assessment. The system consisted of eight infrared Vantage V5 cameras arranged peripherally around the laboratory to ensure complete visibility of the capture volume. Each camera emits and detects infrared light reflected by passive spherical markers (14 mm diameter) attached to anatomical landmarks according to the Plug-in Gait lower body model (Helen Hayes protocol). The cameras recorded at 120 Hz, synchronously capturing the three-dimensional trajectories of each marker with sub-millimetric precision (<1 mm error). Trajectories were reconstructed using Vicon Nexus software (version 2.16), which applies triangulation algorithms to compute spatial coordinates from multiple camera perspectives. Before each session, static and dynamic calibrations were performed using a reference wand and frame to align the camera coordinate system. During gait trials, participants walked inside the calibrated capture volume while the system automatically tracked the motion of each marker. Trajectories with data gaps shorter than 40 frames were interpolated using pattern or spline algorithms following the recommendations of Davis et al. [35]. The following parameters were extracted: gait velocity (m/s), cadence (steps/min), and range-of-motion (ROM) asymmetry indices for pelvis, hip, knee, and ankle joints.

The asymmetry index (AI) between the right and left limbs was calculated according to Robinson et al. [48], using the following expression:

AI (%) = (|L − R|/(0.5 × (L + R))) × 100

where L represents the range of motion (ROM) of the left limb and R that of the right limb, and a value of 0% indicates perfect symmetry between both sides. The Vicon^®^ Motion Capture System has been validated as a reliable and accurate tool for three-dimensional gait analysis in both healthy individuals and patients with neurological disorders, including MS [49]. Its high spatial and angular resolution supports the precision of the kinematic measurements obtained in the present study.

The following biomechanical variables were then considered: Gait velocity (in meters per second), Cadence (steps per minute), Asymmetry index of single-leg support (%SI), Asymmetry index of total ROM (IART) for all joints (pelvis, hip, knee, and ankle), Asymmetry index of ROM during the stance phase (IARA) for all joints (pelvis, hip, knee, and ankle), and Asymmetry index of ROM during the swing phase (IARO) for all joints (pelvis, hip, knee, and ankle).

### 2.2. Study Procedures

**Figure 1:**
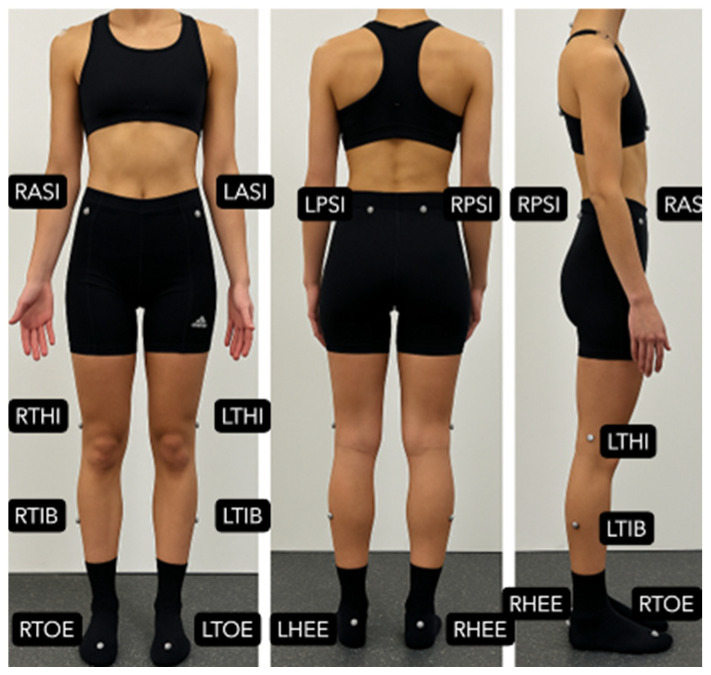
Marker placement according to the modified Plug-in Gait lower body model (Vicon^®^, Oxford Metrics, Oxford, UK) [35]. The figure shows anterior, posterior, and lateral views with pelvic and lower limb markers (LASI, RASI, LPSI, RPSI, LTHI, RTHI, LTIB, RTIB, LHEE, RHEE, LTOE, RTOE).

The assessment sessions were held individually and lasted approximately 30 min. At the beginning of the sessions, the following data were collected: gender, age, type of MS, time of diagnosis, EDSS score (established by the evaluator based on previously described criteria [18], and pharmacological and non-pharmacological treatment. Then, the following clinical variables were assessed: MusiQoL [30,31], Fatigue Scale for Motor and Cognitive Functions (FMSC) [50], Trail Making Test (TMT) A and B [51], and the Berg Balance Scale (BBS) [52]. Afterwards, reflective markers were placed on anatomical landmarks according to the Vicon^®^ Clinical Model (VCM) protocol [35,36], as shown in Figure 1, and the cameras were calibrated. Before starting the recording, perceived fatigue was monitored using the modified Borg Scale [53]. During data acquisition, each participant walked back and forth along a 10-m walkway within the calibrated capture volume of the Vicon^®^ system at a self-selected fast pace. Several consecutive strides were recorded during this continuous trial, and three complete gait cycles per limb were selected from the central portion of the recording, once a steady walking pattern had been reached, to minimize acceleration and deceleration effects. This approach follows previous methodological recommendations for marker-based gait analysis in people with MS [28,34]. These cycles were used to compute the mean kinematic parameters for subsequent analyses.

Assessments were performed by three physiotherapists specialized in neurorehabilitation and gait analysis. Data processing was conducted by an engineer experienced with the Vicon^®^ system, and data analysis by another physiotherapist. Although no formal blinding was applicable, the assessors were unaware of participants’ clinical and questionnaire results during testing, thereby minimizing potential measurement bias.

Each participant attended a single 30-min session, including informed consent, clinical assessment, reflective marker placement, gait recording, and data processing, following the sequence previously described.

### 2.3. Statistical Analysis

IBM SPSS Statistics for Windows, version 29.0 (IBM Corp., Armonk, NY, USA) was used to perform the statistical analysis. Descriptive analysis was performed by determining the mean and standard deviation for quantitative variables and absolute and relative frequencies for categorical variables. The Shapiro–Wilk test was used to test the normality of quantitative variables. Due to the non-normal nature of the data, correlations were performed using Spearman’s correlation coefficient. Following the criterion of Hopkins et al. [54], correlation coefficients were classified as negligible (ρ < 0.1), small (ρ ≥ 0.1 and <0.3), moderate (ρ ≥ 0.3 and <0.5), high (ρ ≥ 0.5 and <0.7), very high (ρ ≥ 0.7 and <0.9) and almost perfect (ρ ≥ 0.9).

No correction for multiple comparisons was applied due to the exploratory nature of the study and the small sample size; *p*-values should therefore be interpreted as indicative. The analysis was designed to explore potential associations within the total sample rather than to perform subgroup comparisons by MS subtype, given that the study was powered based on the overall cohort.

Different linear regression models were applied based on a combined statistical analysis strategy, considering variables that demonstrated a high level of correlation and clinical relevance, with the aim of including the most relevant factors for QoL in patients with MS. The stepwise regression method [55] was used with entry (*p* ≤ 0.05) and removal (*p* ≥ 0.10) criteria, allowing the exclusion of variables that did not contribute significantly to the model or introduced statistical redundancy (assessed via tolerance and variance inflation factor, VIF). Additionally, the coefficient of determination (R^2^) was used to evaluate the explanatory power of the model, and the Durbin-Watson statistic was applied to ensure that model assumptions were met.

Statistical significance was set at *p* ≤ 0.05.

## 3. Results

A total of 32 patients were included in the study, of whom 22 (68.75%) were women. The RRMS was the most prevalent type of MS, representing 78.1% of cases (n = 25), followed by the PPMS form (18.8%; n = 6), and the SPMS (3.1%; n = 1). The mean age of participants was 45 ± 10.14 years, and the mean EDSS score was 3.81 ± 1.56 points. Table 1 shows the descriptive analysis of study variables. The mean MusiQoL-31 score was 65.68 ± 15.20 points.

Correlations among variables

**Figure 2:**
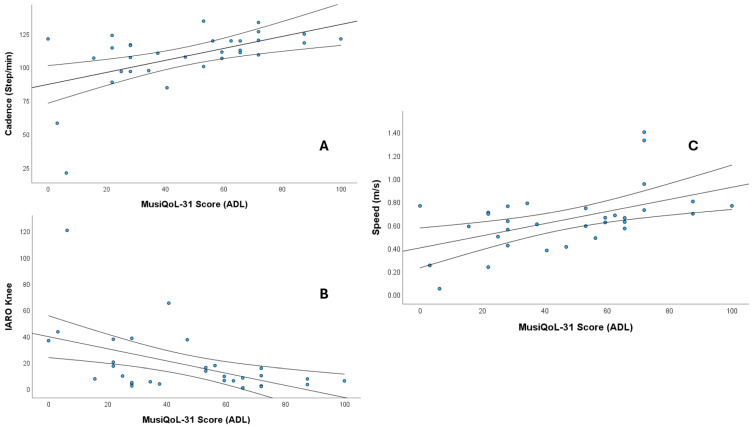
Scatter plots showing the significant correlations (Spearman’s ρ > 0.5) between MusiQoL-31 Activities of Daily Living (ADL) scores and gait parameters. (**A**) Cadence (steps/min), (**B**) IARO knee, and (**C**) Speed (m/s). The central line represents the regression line, and the outer lines represent the confidence intervals.

The ADL dimension showed strong correlations with cadence (ρ = 0.508), speed (ρ = 0.506), and knee IARO (ρ = −0.525) (*p* < 0.01). Additional significant moderate correlations were observed between cadence and REJ (ρ = 0.422) and the global score (ρ = 0.383); and RHCS (ρ = 0.453); hip IART with REJ (ρ = −0.411); knee IART with RFa (ρ = 0.411); hip IARA with REJ (ρ = −0.362); knee IARA with PWB (ρ = −0.397); and knee IARO with RFr (ρ = 0.411) and RHCS (ρ = 0.458) (all *p* < 0.05). Figure 2 shows scatter plots showing the significant correlations (ρ > 0.5).

Fatigue was strongly associated with quality of life: very high correlations were found between SPT and cognitive FSMC (ρ = −0.796) and total FSMC (ρ = −0.775), as well as between ADL and motor FSMC (ρ = −0.708) (*p* < 0.05). High correlations (*p* < 0.01) were also observed between cognitive FSMC and the global score (ρ = −0.628); motor FSMC and SPT (ρ = −0.656); total FSMC with ADL (ρ = −0.550) and the global score (ρ = −0.551); and the Borg scale with ADL (ρ = −0.547).

Regarding clinical variables related to attention and executive functions, a high-intensity correlation (*p* < 0.01) was observed between TMT-B and ADL (ρ = −0.517). Additionally, TMT-A showed moderate-intensity correlations with ADL (ρ = −0.448) and SPT (ρ = −0.421) dimensions, as well as with the global score (ρ = −0.359); these correlations were statistically significant (*p* < 0.05).

**Figure 3:**
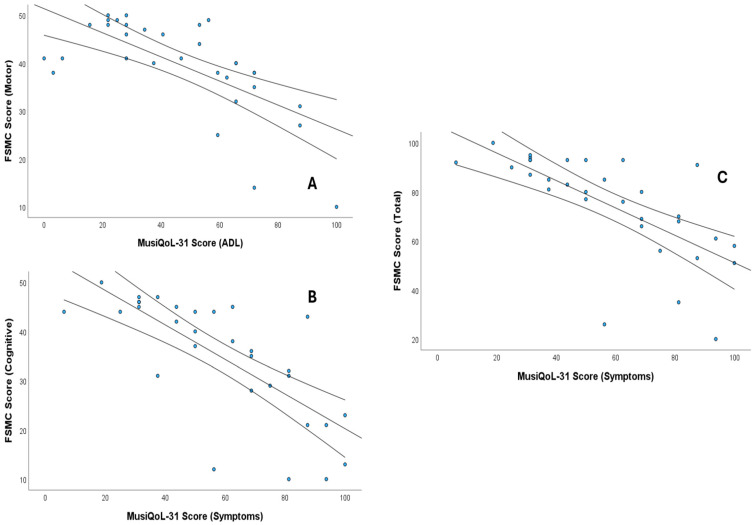
Scatter plots showing the significant correlations (Spearman’s ρ > 0.7) between MusiQoL-31 scores and fatigue measures (FSMC). (**A**) Correlation between MusiQoL-31 Activities of Daily Living (ADL) and FSMC Motor Score, (**B**) Correlation between MusiQoL-31 Symptoms and FSMC Cognitive Score, and (**C**) Correlation between MusiQoL-31 Symptoms and FSMC Total Score. The central line represents the regression line, and the outer lines represent the confidence intervals.

Finally, balance (BBS) and EDSS correlated at a high-intensity level with ADL (ρ = 0.635 and ρ = −0.628, respectively) (*p* < 0.01). Likewise, ADL showed a moderate correlation with age (ρ = −0.365) (*p* < 0.05). Figure 3 shows scatter plots showing the significant correlations (ρ > 0.7). In the Supplemental Material, Tables S1 and S2 show the values and significance of all correlations performed.

Linear Regression models

The regression analysis indicated that the combination of TMT-A (β = −0.398, 95% CI −0.746, −0.051) and cognitive FSMC (β = −0.456, 95% CI −0.869, −0.042) significantly predicted the global score of the MusiQoL-31 questionnaire, explaining 35% of the total variance (R^2^ = 0.392, adjusted R^2^ = 0.350, F(2,29) = 9.357, *p* < 0.001). Higher TMT-A times (slower processing and attention) and greater cognitive fatigue were associated with lower overall quality-of-life scores.

The model developed for the ADL dimension, which included motor FSMC (β = −1.488, 95% CI −2.167, −0.810) and BBS (β = 1.201, 95% CI 0.404, 1.998), explained 55.5% of the variance (R^2^ = 0.584; adjusted R^2^ = 0.555; F(2,29) = 20.323, *p* < 0.001). Motor FSMC was negatively associated with ADL scores, whereas higher balance (BBS) scores were associated with higher ADL scores.

Finally, the model for the REJ dimension revealed that hip IARA (β = −0.703, 95% CI −1.130, −0.277) was a significant predictor of this subscale, explaining 25% of the variance (R^2^ = 0.274, adjusted R^2^ = 0.250, F(1,30) = 11.336, *p* = 0.002).

All models showed independence of residuals (Durbin-Watson values between 1.7 and 2.2) and did not present multicollinearity issues (VIF < 1.1).

## 4. Discussion

The findings of this study indicate that health-related QoL, assessed with the MusiQoL-31, is significantly associated with biomechanical gait parameters and with clinical factors such as fatigue, postural balance, and cognitive performance in individuals with MS.

The observation that gait speed and cadence correlate positively with the ADL dimension is consistent with previous research identifying these parameters as key predictors of functional status and fall risk in this population [56,57,58,59]. For instance, Pau et al. reported that reduced gait speed and the presence of kinematic asymmetries were associated with greater functional deterioration [28]. Our data reinforce this evidence by showing that joint asymmetry—specifically the knee asymmetry measured by the Gait Relative Asymmetry Index—is also related to specific aspects of QoL, including both the ability to perform ADL and the perception of social rejection.

Fatigue, in both its motor and cognitive components, exhibited the strongest correlations with the various dimensions assessed by the MusiQoL questionnaire. This finding aligns with existing evidence describing fatigue as one of the most frequent and disabling symptoms of MS, profoundly impacting functional independence as well as physical and mental performance [60]. As noted by Weiland et al. [20], the presence of fatigue is associated with poorer performance in daily activities and an increased risk of falls, both of which directly affect perceived well-being. In line with these findings, our results revealed a marked negative relationship between motor fatigue scores and the ability to carry out daily tasks independently, underscoring the need to address this symptom comprehensively in clinical practice.

Regarding balance, our results show that scores on the BBS are positively correlated with the ability to manage daily tasks. This observation is consistent with the findings of Fritz et al. [22], who demonstrated that dynamic balance has a significant impact on gait speed and overall functional capacity. Clinically, this can be explained by the fact that better postural control enables safer transitions during ambulation—such as turning, changing direction, or adapting to uneven surfaces—thereby enhancing autonomy and reducing the risk of falls in everyday environments.

Furthermore, the time required to complete the TMT was associated with lower QoL scores. This result aligns with prior studies highlighting the role of cognitive slowing in the planning and execution of functional activities [17,29]. From a clinical perspective, reduced processing speed and diminished cognitive flexibility make it more difficult to organize motor sequences and make rapid decisions, increasing the mental effort required for routine tasks. This, in turn, can lead to frustration, greater dependency, and ultimately a reduced perception of well-being.

Interestingly, knee joint asymmetry was also associated with the social rejection dimension. While previous studies have focused mainly on the functional implications of gait asymmetry, particularly its association with disability and fall risk [19,28], its potential psychosocial impact has received little attention. In this regard, the present study contributes by linking an objective kinematic parameter to a subjective domain of QoL, suggesting that visible gait alterations may influence self-perception and social interactions, thereby contributing to feelings of stigma or social withdrawal [38,39].

The identification of specific kinematic parameters linked to different aspects of QoL suggests that rehabilitation approaches for individuals with MS should not only consider gait speed and cadence but also gait symmetry and the cognitive and emotional factors influencing motor performance. From a clinical perspective, a deeper understanding of how these gait characteristics interact with fatigue, balance, and cognitive function can help refine therapeutic strategies and make them more individualized. The use of three-dimensional motion capture systems such as Vicon^®^ contributes to this goal by providing precise, objective data that improve our understanding of gait alterations and their impact on functional independence and well-being.

### Limitations

The cross-sectional design of this study limits the ability to establish causal relationships between the variables analyzed. In addition, the sample included individuals with different clinical forms of MS, and there was no control group for comparison with healthy subjects or other populations. Assessments were conducted under controlled laboratory conditions, which may limit the generalizability of the findings to real-world settings, and external variables such as ongoing pharmacological treatments were not considered. Moreover, the relatively small sample size (n = 32), predominantly composed of women, and the inclusion of heterogeneous clinical phenotypes (RRMS, PPMS, SPMS) may have introduced variability and limited the possibility of performing subgroup analyses by sex or MS type without compromising statistical power. However, the use of a validated and reliable optical motion capture system (Vicon^®^) contributes to the methodological consistency of the kinematic data.

In addition, as the study included participants who were able to walk independently or with the aid of assistive devices, the findings may not be generalizable to individuals with more advanced disability or those unable to perform gait assessments. As this was an exploratory study involving multiple correlations, the results should be interpreted cautiously, as they require confirmation in larger samples. Nevertheless, the results are largely consistent with previous studies and provide a solid foundation for future longitudinal research with greater statistical power to further explore the relationships between biomechanical parameters, clinical factors, and QoL in this population.

## 5. Conclusions

QoL in individuals with MS is strongly influenced by both biomechanical gait parameters and clinical factors such as fatigue, postural balance, and cognitive performance. Specifically, gait speed, cadence, and knee joint symmetry were associated with the ability to perform ADL, while motor and cognitive fatigue emerged as the most relevant predictors of reduced overall well-being. Moreover, executive function and postural stability played a decisive role in functional autonomy. Importantly, regression models explained up to 58.4% of the variance in the ADL dimension, underscoring that these combined factors account for a substantial proportion of QoL outcomes. These findings highlight the importance of comprehensive assessments to better inform physiotherapeutic interventions aimed at improving daily functioning and overall QoL in this population.

## Figures and Tables

**Table 1:** Descriptive statistics of dependent and independent variables (Mean ± SD) (minimum, maximum).

Variable	Mean ± SD
Age (years)	45.12 ± 10.14 (27–67)
MusiQoL-31-ADL	46.88 ± 26.55 (0–100)
MusiQoL-31-A PWB	46.63 ± 25.99 (0–100)
MusiQoL-31-A SPT	54.33 ± 25.36 (6.25–100)
MusiQoL-31-A RFr	68.27 ± 28.87 (0–100)
MusiQoL-31-A RFa	82.05 ± 26.11 (16.67–100)
MusiQoL-31-A RHCS	81.41 ± 26.70 (0–100)
MusiQoL-31-A SSL	76.92 ± 26.15 (12.50–100)
MusiQoL-31-A COP	56.73 ± 31.27 (0–100)
MusiQoL-31-A REJ	77.88 ± 24.83 (25–100)
MusiQoL-31-Global	65.68 ± 15.20 (36.92–95.49)
EDSS	3.81 ± 1.56 (1–6.5)
FSMC cognitive	36.04 ± 11.89 (10–50)
FSMC motor	39.81 ± 10.27 (10–62)
FSMC total	75.85 ± 20.86(20–100)
BORG scale	1.35 ± 0.37 (0–4)
TMT-A	38.27 ± 15.45 (16–102)
TMT-B	85.31 ± 50.81 (33–301)
BBS	50.85 ± 8.29 (22–56)
Cadence (steps/min)	110.51 ± 15.57 (20.8–141.23)
Velocity (m/s)	0.66 ± 0.24 (0.05–1.75)

Abbreviations: MusiQoL-31, Multiple Sclerosis International Quality of Life Questionnaire, 31 items; ADL, Activities of Daily Living; PWB, Psychological Well-Being; SPT, Symptoms; RFr, Relationships with Friends; RFa, Relationships with Family; RHCS, Relationships with Healthcare System; SSL, Sentimental and Sexual Life; COP, Coping; REJ, Rejection; EDSS, Expanded Disability Status Scale; FSMC, Fatigue Scale for Motor and Cognitive Functions; BORG, Borg Rating of Perceived Exertion Scale; TMT-A, Trail Making Test Part A; TMT-B, Trail Making Test Part B; BBS, Berg Balance Scale.

## Data Availability

Datasets supporting reported results are available upon request to the first author.

## Supplementary Materials

The following supporting information can be downloaded at: https://www.mdpi.com/article/10.3390/s25226909/s1, Table S1: Correlations between MusiQoL-31 and its dimensions and the biomechanical variables. Table S2: Correlations between MusiQoL dimensions and independent variables.
