# Supplementary material for: Gait Kinematics Assessed by Vicon® and Quality of Life Correlations in Multiple Sclerosis Patients: A Cross-Sectional Study
Source: Sensors (Basel). 2025 Nov 12;25(22):6909. doi: 10.3390/s25226909 (PMC12656241; doi:10.3390/s25226909)
Supplement: Supplementary file 1 [file sensors-25-06909-s001.zip › sensors-3925175-supplementary.pdf]

Table S1. Correlations between MusiQoL-31 and its dimensions and the biomechanical variables.

Variable	ADL	PWB	SPT	RFr	RFa	RHCS	SSL	COP	REJ	Global
**Cadence**	0.508 / **0.003**	0.056 / 0.763	0.156 / 0.393	0.058 / 0.753	0.027 / 0.882	0.204 / 0.263	0.141 / 0.440	0.324 / 0.070	0.422 / **0.016**	0.383 / **0.005**
**Velocity**	0.506 / **0.003**	0.079 / 0.668	0.102 / 0.578	0.029 / 0.873	–0.103 / 0.574	-0.001 / 0.994	0.016 / 0.931	0.000 / 0.999	0.175 / 0.074	0.175 / 0.339
**IA SIGLES**	–0.053 / 0.786	0.429 / **0.020**	0.168 / 0.385	0.340 / 0.071	0.136 / 0.483	0.453 / **0.014**	0.106 / 0.585	-0.059 / 0.761	0.052 / 0.788	0.244 / 0.202
**IART pelvis**	–0.188 / 0.302	–0.187 / 0.305	–0.218 / 0.231	0.005 / 0.979	0.018 / 0.924	–0.085 / 0.645	0.047 / 0.798	0.147 / 0.421	0.098 / 0.593	–0.078 / 0.671
**IART hip**	–0.129 / 0.480	–0.021 / 0.911	0.035 / 0.850	0.123 / 0.502	0.160 / 0.383	0.267 / 0.139	0.016 / 0.926	0.017 / 0.926	–0.411 / **0.016**	0.030 / 0.989
**IART knee**	–0.288 / 0.110	0.055 / 0.765	–0.162 / 0.377	0.304 / 0.090	0.411 / **0.020**	0.120 / 0.513	0.290 / 0.108	–0.129 / 0.483	-0.185 / 0.311	0.008 / 0.989
**IART ankle**	–0.022 / 0.907	–0.323 / 0.071	–0.001 / 0.996	–0.016 / 0.929	–0.242 / 0.183	–0.145 / 0.429	0.173 / 0.345	–0.016 / 0.739	–0.269 / 0.137	–0.065 / 0.726
**IARA pelvis**	0.078 / 0.672	0.122 / 0.505	0.102 / 0.580	–0.291 / 0.106	0.225 / 0.216	0.035 / 0.850	0.157 / 0.392	–0.016 / 0.931	–0.157 / 0.391	0.025 / 0.891
**IARA hip**	–0.155 / 0.398	–0.085 / 0.643	–0.057 / 0.757	0.026 / 0.887	0.025 / 0.890	0.170 / 0.353	-0.215 / 0.238	0.109 / 0.554	-.0362 / 0.042	–0.045 / 0.806
**IARA knee**	–0.190 / 0.297	–0.397 / **0.025**	–0.297 / 0.099	–0.163 / 0.372	–0.187 / 0.305	–0.104 / 0.573	–0.124 / 0.498	–0.187 / 0.306	–0.173 / **0.034**	–0.309 / 0.085
**IARA ankle**	–0.021 / 0.909	–0.166 / 0.365	-0.170 / 0.351	0.022 / 0.906	0.181 / 0.322	-0.198 / 0.278	0.208 / 0.252	–0.218 / 0.231	–0.293 / 0.104	–0.141 / 0.442
**IARO pelvis**	–0.120 / 0.514	–0.038 / 0.837	–0.094 / 0.607	–0.032 / 0.862	0.052 / 0.776	0.087 / 0.636	–0.146 / 0.425	0.020 / 0.914	–0.119 / 0.517	–0.020 / 0.923
**IARO hip**	0.044 / 0.814	0.139 / 0.448	0.055 / 0.765	0.192 / 0.293	0.172 / 0.347	0.290 / 0.108	0.169 / 0.355	–0.194 / 0.287	–0.150 / 0.412	0.112 / 0.542
**IARO knee**	–0.525 / **0.002**	–0.106 / 0.563	–0.330 / **0.030**	0.411 / **0.019**	0.458 / **0.008**	0.047 / 0.797	0.347 / 0.052	0.005 / 0.978	-0.003 / 0.986	0.111 / 0.544
**IARO ankle**	-0.011 / 0.954	–0.141 / 0.441	0.281 / 0.120	0.138 / 0.451	0.113 / 0.539	0.290 / 0.096	0.124 / 0.497	0.026 / 0.889	-0.250 / 0.168	0.185 / 0.310

Data are shown as ρ / p-value. Abbreviations: ADL: activities of daily living; PWB: psychological well-being; SPT: symptoms; RFr: relationship with friends; RFa: relationship with family; RHCS: relationship with the healthcare system; SSL: sentimental and sexual life; COP: coping; REJ: rejection; %SI: Single-leg support percentage asymmetry index; IART: Total range of motion asymmetry index; IARA: Stance phase range of motion asymmetry index; IARO: Swing phase range of motion asymmetry index

Table S2. Correlations between MusiQoL dimensions and independent variables

Variable	ADL	PWB	SPT	RFr	RFa	RHCS	SSL	COP	REJ	Global
**FSMC cognitive**	–0.404 / **0.022**	–0.487 / **0.005**	–0.796 / **0.000**	–0.288 / 0.110	–0.375 / **0.034**	–0.084 / 0.649	–0.321 / 0.074	–0.306 / 0.088	–0.356 / **0.046**	–0.628 / **0.000**
**FSMC motor**	–0.708 / **0.000**	–0.438 / **0.012**	–0.656 / **0.000**	–0.125 / 0.495	–0.159 / 0.385	–0.046 / 0.801	–0.049 / 0.789	–0.151 / 0.410	–0.159 / 0.384	–0.432 / **0.013**
**FSMC total**	–0.550 / **0.001**	–0.466 / **0.007**	–0.775 / **0.000**	–0.228 / 0.209	–0.286 / 0.113	–0.065 / 0.725	–0.194 / 0.287	–0.233 / 0.199	–0.258 / 0.153	–0.551 / **0.001**
**TMT-A**	–0.448 / **0.010**	–0.086 / 0.639	–0.421 / **0.016**	0.094 / 0.680	–0.076 / 0.739	–0.162 / 0.362	–0.260 / 0.150	–0.256 / 0.158	0.153 / 0.403	–0.359 / **0.034**
**TMT-B**	–0.517 / **0.002**	–0.140 / 0.445	–0.244 / 0.178	0.197 / 0.279	–0.092 / 0.617	–0.122 / 0.506	–0.208 / 0.252	–0.189 / 0.300	0.452 / **0.006**	–0.245 / 0.176
**BBS**	0.635 / **0.000**	–0.015 / 0.935	0.218 / 0.230	–0.110 / 0.551	–0.080 / 0.664	0.049 / 0.792	0.180 / 0.324	0.124 / 0.498	0.338 / **0.020**	0.201 / 0.180
**Age**	–0.365 / **0.040**	0.287 / 0.112	–0.070 / 0.703	0.074 / 0.689	–0.002 / 0.993	0.132 / 0.471	–0.072 / 0.694	0.181 / 0.320	0.083 / 0.658	–0.063 / 0.843
**EDSS**	–0.628 / **0.000**	0.123 / 0.519	–0.157 / 0.409	0.124 / 0.514	0.053 / 0.768	0.114 / 0.548	0.082 / 0.627	0.563 / **0.000**	–0.182 / 0.363	–0.128 / 0.333
**BORG**	–0.546 / **0.001**	–0.161 / 0.377	–0.137 / 0.454	0.255 / 0.195	0.028 / 0.877	–0.055 / 0.763	–0.125 / 0.495	–0.045 / 0.807	0.329 / **0.039**	–0.177 / 0.333

Data are shown as ρ / p-value. Abbreviations: MusiQoL-31, Multiple Sclerosis International Quality of Life Questionnaire, 31 items; ADL, Activities of Daily Living; PWB, Psychological Well-Being; SPT, Symptoms; RFr, Relationships with Friends; RFa, Relationships with Family; RHCS, Relationships with Healthcare System; SSL, Sentimental and Sexual Life; COP, Coping; REJ, Rejection; FSMC, Fatigue Scale for Motor and Cognitive Functions; TMT-A, Trail Making Test Part A; TMT-B, Trail Making Test Part B; BBS, Berg Balance Scale; EDSS, Expanded Disability Status Scale; BORG, Borg Rating of Perceived Exertion Scale
